# Synthesis of vibroarthrographic signals in knee osteoarthritis diagnosis training

**DOI:** 10.1186/s13104-016-2156-6

**Published:** 2016-07-19

**Authors:** Chin-Shiuh Shieh, Chin-Dar Tseng, Li-Yun Chang, Wei-Chun Lin, Li-Fu Wu, Hung-Yu Wang, Pei-Ju Chao, Chien-Liang Chiu, Tsair-Fwu Lee

**Affiliations:** Medical Physics and Informatics Laboratory of Electronics Engineering, National Kaohsiung University of Applied Sciences, 415, Chien Kung Road, San-Min District, Kaohsiung, 807 Taiwan, ROC; Graduate Institute of Clinical Medicine, Kaohsiung Medical University, Kaohsiung, 807 Taiwan, ROC; Department of Radiation Oncology, Kaohsiung Chang Gung Memorial Hospital and Chang Gung University College of Medicine, Kaohsiung, 83342 Taiwan, ROC; Department of Medical Imaging and Radiological Sciences, I-Shou University, Kaohsiung, 82445 Taiwan, ROC; Institute of Photonics and Communications, National Kaohsiung University of Applied Sciences, Kaohsiung, 80778 Taiwan, ROC; Department of Orthopedic, Kaohsiung Municipal Min-Sheng Hospital, Kaohsiung, 80276 Taiwan, ROC

**Keywords:** Vibroarthrographic signals, Knee sound synthesis, Kellgren–Lawrence grading

## Abstract

**Background:**

Vibroarthrographic (VAG) signals are used as useful indicators of knee osteoarthritis (OA) status. The objective was to build a template database of knee crepitus sounds. Internships can practice in the template database to shorten the time of training for diagnosis of OA.

**Methods:**

A knee sound signal was obtained using an innovative stethoscope device with a goniometer. Each knee sound signal was recorded with a Kellgren–Lawrence (KL) grade. The sound signal was segmented according to the goniometer data. The signal was Fourier transformed on the correlated frequency segment. An inverse Fourier transform was performed to obtain the time-domain signal. Haar wavelet transform was then done. The median and mean of the wavelet coefficients were chosen to inverse transform the synthesized signal in each KL category. The quality of the synthesized signal was assessed by a clinician.

**Results:**

The sample signals were evaluated using different algorithms (median and mean). The accuracy rate of the median coefficient algorithm (93 %) was better than the mean coefficient algorithm (88 %) for cross-validation by a clinician using synthesis of VAG.

**Conclusions:**

The artificial signal we synthesized has the potential to build a learning system for medical students, internships and para-medical personnel for the diagnosis of OA. Therefore, our method provides a feasible way to evaluate crepitus sounds that may assist in the diagnosis of knee OA.

## Background

Osteoarthritis (OA) is a progressive degradation process that can occur in a number of joints. OA causes low-grade inflammation in joints and mainly affects the elderly [1, 2]. Approximately 30 % of those over the age of 65 suffer from OA. Its pathological signs include joint angulation [3], osteophyte formation, and joint space narrowing [4, 5].

Most common non-traumatic knee idiopathic conditions, including on known as the patella cartilage fibrillates [6], softening, and beneath the surface of patella off [7–9]. Unless there is a significant symptomatic or anatomical hypertrophy, identifying articular cartilage changes is very difficult. Imaging techniques, such as computed tomography (CT), X-ray imaging, and magnetic resonance imaging (MRI) scans, offer some hope for non-invasive detection of cartilage pathologies [10]. It is impractical to display the functional integrity of cartilage just in terms of its stiffness, softening, or fissuring [11].

Arthroscopy is a traditional method for the evaluation of cartilage conditions in the pathological diagnosis of cartilage. However, arthroscopy often causes side effects because it is intrusive. There are some of the risks of surgery and anesthesia. Repeated arthroscopic evaluation of patients would not be practical [12, 13].

During clinical observation, the knee of patients with OA often exhibits a crepitus phenomenon [14]. Using one hand placed on the patella, the examiner performs the physical examination during the passive range of motion (ROM). However, this observation is a very subjective experience and cannot be recorded for analytical purposes [15]. Crepitus alone is not an indication of osteoarthritis, and is only one of several indicators in published criteria [16].

Foregoing dysfunctions of the knee joint sound auscultation by analyzing the non-invasive diagnosis, many researchers have reported that the feasibility and effectiveness of this technology [17]. Collecting audio signals recorded on the knee, Chu et al. developed good methods in order to reduce environmental noise such as background and skin friction, the signal is divided into different categories [18, 19]. Kernohan et al. stated that coverage of 86 % of a meniscal injury would produce characteristic signals, and normal changes combined with swelling may be useful pointers of early cartilage degeneration [20]. McCoy et al. showed that meniscus injuries and arthritis knee sound analysis, based on sound peaks dividing frequency groups, can be used for the identification of chondromalacia patellar [21].

Various knee sound signal processing techniques have been carried out under joint pathology classification of normal and abnormal test the knee, such as autoregression [22, 23], least square [24], and linear prediction modeling [25, 26], as well as time–frequency analysis [27] and wavelet decomposition [28, 29]. The setup of the system for acquiring vibroarthrographic (VAG) signals was proposed by Lin et al. [30–32].

In this study, we aimed to synthesize the knee sounds in vitro based on the frequency components obtained from a previous study [33]. The objective of this paper was to build a template database for knee crepitus sound. Because the sound and vibration of OA can be reproduced through this method, learners of diagnosing OA immediately familiarize the sound and vibration made by OA. In this study, we aimed to synthesize the knee sounds in vitro based on the frequency components obtained from a previous study to help generate joint sounds useful for teaching. This method will replace or shorten the training of less experienced orthopedists or internships for the diagnosis of OA. To the best of our knowledge, there have been no previous studies concerning the in vitro synthesis of crepitus sounds for knee OA. Previous studies have concentrated on the frequency parts of the signal, i.e., the analytical approach for signal processing. However, the reverse way, i.e., a synthetic approach, is explored in this study. Once the sound model database was built, the usefulness for OA diagnosis was then explored.

## Methods

### Demographic data

Fifty-nine patients with degenerative arthritis and 85 patients with no history of knee injury or discomfort were enrolled. Their demographic data, including age, sex, and disease status, were recorded. Every OA patient had a knee X-ray for anterior-posterior and lateral views for disease grading with Kellgren–Lawrence (KL) scores by two independent orthopedic surgeons. The KL grades were recorded for three partitions of the knee joint: lateral, medial, and patellofemoral (PF). The KL grading scale is based on the state of OA in five grades, with 0 indicating normal and 4 indicating the most severe manifestation of the disease.

The KL grading ranges from 0–4: Grade 0: none (definitely no OA); Grade 1: doubtful (doubtful possible osteophytes or narrowing of joint space); Grade 2: minimal (definite osteophytes); Grade 3: moderate (moderate osteophytes, definite slight sclerosis, narrowing of joint space, and possible deformity of bone contours); Grade 4: severe (serious osteophytes, outstanding narrowing of joint space, serious sclerosis, and definite deformity of bone contours). Since the knee sound signals might be changed by the pressure between the bell of the stethoscope and the skin, a specially fitted knee brace was used to hold the stethoscope in front of the patella. The KL grading was used for the comparison.

### Experimental protocol

The electro-stethoscope was assembled with a conventional stethoscope and a high-performance microphone. The microphone was connected to the line-in port of a personal computer (PC) for obtaining sound signals. The sound signals were transferred by the PC. Each recorded sampling criteria was 44,100 Hz and 16 bits per sample rate for digitizing. A scalable electro-goniometer was set on the side of the knee for measuring joint rotation. The pressure between the skin and the bell of the stethoscope may change the knee sound signals, so a suitable knee brace was used to grip the stethoscope in front of the patella [30].

The contact zone selected for auscultation on the condyle of patellar position was considered physiological structure and location so that we could eliminate the disturbance of muscle contraction. The knee joint in the flexion position is defined as 90º and the knee in full extension is defined as 0º. One ROM cycle contained more than the approximate angle 90° → 0° → 90° range of one passive flexion and passive extension within a period of 2 s. The knee joint was moved passively by an examiner between 0 and 90°, using the electro-goniometer as a guide. The examiner attempted to maintain a constant angular velocity during the process of manually moving the joint.

**Fig. 1:**
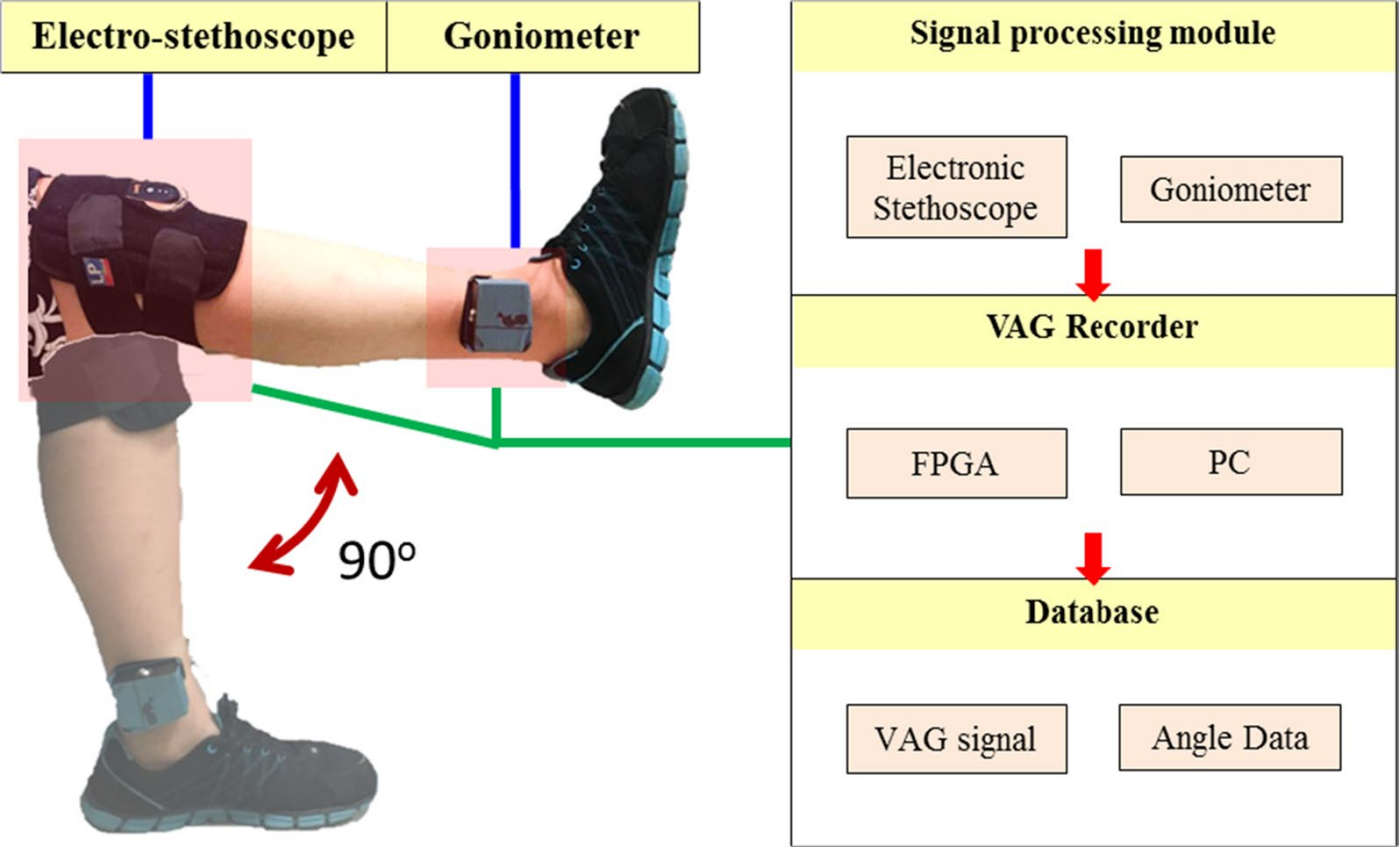
Knee flexed (90°) and fully extended (0°) in a sitting position as measured with a goniometer. *FPGA* Field Programmable Gate Array

The subject was warmed up passively for maintaining the speed of knee flexion and extension constant. After some ROM cycles training in the sitting position, each subject underwent passive ROM cycles for 20 s, maintaining a constant angular velocity. During data collection, the passive ROM cycle was remained to ~2 s in each cycle. If the collection cycle is not exactly of the required length, the changes might affect the sound signals of the angular velocity of the knee, which would lead to the acquisition of signal frequency characteristic error. Therefore, in order to minimize such things from happening, the sound signals obtained in a single cycle were divided according to the knee joint angle that was recorded by the goniometer. In Fig. 1, knee flexed (90°) and fully extended (0°) in a sitting position as measured with a goniometer.

In Fig. 3, the sound signals are arranged in one column with the goniometer data. In 25–75° of flexion process of collecting sound signals are used for further analysis. Corresponding to the start and end stages of the knee ROM, respectively, if there is too much noise, the signals during 0–25° and 75–90° will be discarded. From the physio-anatomical point of view, at the start and end stages of the knee ROM, the patella does not make full contact with the surface of the femur. In addition, there is a lot of background noise related to the acceleration/deceleration of the knee joint during such a period. Consequently, only the segment of 25–75° was preserved. In Fig. 3, the ascending signal limb is tagged “up” and the descending limb is tagged “down”. The ascending limb indicates the knee extension, and the descending limb indicates the knee flexion. Although the two limbs have the same tracking route, the patella on the femur surface can still be seen the differences.

### Signal processing and feature selection

The sound signal in the time domain is input as a computer analysis as standard vector. A fast Fourier transform of the signal was carried out. A ratio index ($R$) was designed to characterize the power spectrum property of the signals:

1 $$ R = \frac{{\int_{{l_{1} }}^{{u_{1} }} {f\left( x \right)^{2} } dx}}{{\int_{l}^{u} {f\left( x \right)^{2} } dx}} $$

In Eq. (1), the lower limit *l* is set as 5 for integration, and the upper limit *u* is set as 500. The denominator is the partial sum of the power spectrum of the signal in the frequency domain. There are two reasons why the lower limit *l* cannot be 1.

First, the low-frequency value of 1 means DC component, and can be seen the roaming of the baseline is a phenomenon highlighted by sound signal. Secondly, the first 4 cycles of low frequency components may cause interference with the knee ROM cycle. Therefore, the partial sum of the power spectrum must start from *l* = 5. In contrast, the choice of the upper limit *u* is more flexible. In the low frequency region, Fourier energy spectrum has greater concentration.

In Eq. (1), the denominator is designed to be representative of the signal variability between the normal and osteoarthritic knee. The lower limit $l_{1}$ of this integration is set to 20 and the upper limit $u_{1}$ is set to 100.

Every patient can get 10 different $R$ values, which form the sample space. In our previous study, the $R$ numeric is the basis for further disease classification studies. However, in this work, we wished to seek the frequency segment that had the highest correlation with KL grades:

2 $$ R_{i} = \frac{{\int_{{s_{i} }}^{{s_{i} + 10}} {f\left( x \right)^{2} } dx}}{{\int_{l}^{u} {f\left( x \right)^{2} } dx}} $$

In Eq. (2), the denominator is kept the same as in Eq. (1). However, the integration in the denominator is different from Eq. (1). The integration lower limit $S_{i}$ is taken from 10 to 500 with a step of 10. That is, for every single signal, a different frequency interval is calculated for its $R_{i}$ value. In this study, we called this value the “segmented $R$”, or $R_{i}$. This value is then correlated with KL grades, using the formula in Eq. (3):

3 $$ r = \frac{{\sum\nolimits_{i = 1}^{n} {\left( {X_{i} - \bar{X}} \right)\left( {Y_{i} - \bar{Y}} \right)} }}{{\sqrt {\sum\nolimits_{i = 1}^{n} {\left( {X_{i} - \bar{X}} \right)^{2} } } \sqrt {\sum\nolimits_{i = 1}^{n} {\left( {Y_{i} - \bar{Y}} \right)^{2} } } }} $$

### Artificial signal synthesis

#### Phase 1: collection of VAG sounds from patients

In preliminary results, the highest correlated frequency segments were found to be around 50–150 Hz. If the most highly correlated frequency segments can be defined, then we can detect the corresponding Fourier coefficients from the subjects, i.e., we will form a vector of length 10 for each individual for every frequency segment.

#### Phase 2: synthesis of in vitro sounds for a training database

To eliminate the influence of noise and the outliers, the median of this vector element was used as the desired weighting. Although the Fourier coefficient may be complex in nature, we attempted to handle this problem by separation of real and complex parts, and then this coefficient is used to synthesize the sound signal. The sound signal is then extended to 20 s.

#### Phase 3: reliability assessment of the synthesized sounds

The synthesized sound is outputted to a highly sensitive speaker for further evaluation. A knee cast was built with a pant cloth covering on it. The speaker was placed under the patella surface. The sound was played with the examiner’s hand placed over the patella surface. For assessing the quality of the signal we synthesized, we performed a questionnaire containing the Likert scale to evaluate the quality of the sound. The questionnaire was answered by three independent orthopedic surgeons. The descriptive statistics and analysis was carried out using SPSS software.

### Classification based on synthetic sound signals

Eventually, the database representing every patient and normal subjects was built. If there was an unknown signal to be classified, the decision rule was based on the distance defined by the Kullback–Leibler divergence (KL divergence), which is a metric used in information theory and it is non-symmetric in nature. However, it can provide a convenient and elegant way of measuring the similarity of two discrete probability distribution functions. The KL divergence for discrete probability distribution was calculated as in Eq. (4):

4 $$ D_{KL} \left( {P,Q} \right) = \sum\limits_{i = 1} {\ln \left( {\frac{P\left( i \right)}{Q\left( i \right)}} \right)} P\left( i \right) $$

where $P\left( i \right)$ represents the unknown signal in normalized vector form, and $Q\left( i \right)$ is the patient’s synthetic sound signal. The classification process is thereafter a simple task: pick the smallest KL divergence, and the KL grade belonging to the signal is then the OA diagnosis of the unknown signal. Based on this method, the classification accuracy rate is then calculated with a leave-one-out sampling method.

## Results

**Fig. 2:**
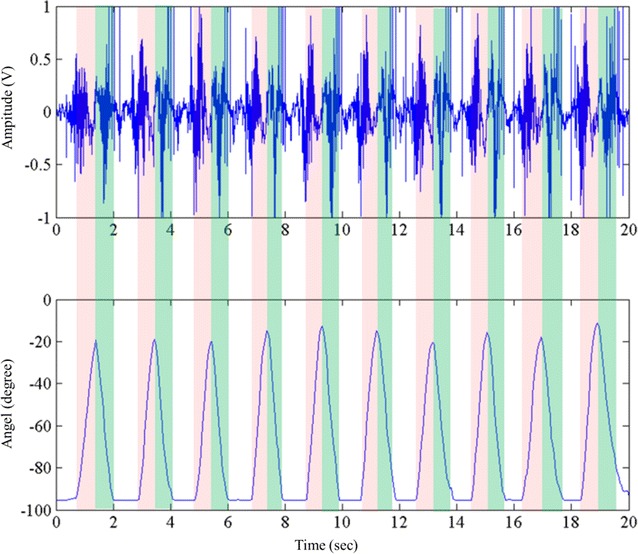
One sample of knee sound signal acquired. The sound amplitude data is aligned with goniometer data. The data is composed of 8 cycles. Each cycle can be segmented into ascending and descending limbs

**Fig. 3:**
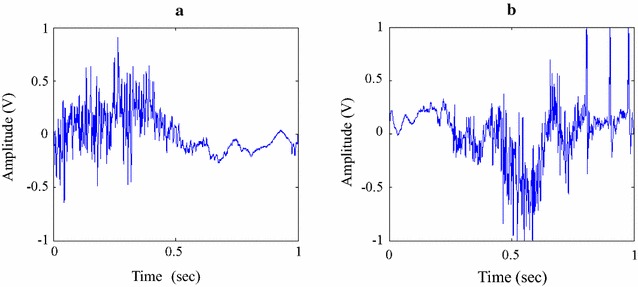
The detailed view of ascending and descending limbs is shown in **a**, **b**. There is some similarity between the two waveforms. However, they are not all the same due to inhomogeneous process of knee extension and flexion

The system for VAG sound signal collection was shown in an earlier report [30]. The number of patients for sound signal collection was 144, which were grouped by gender (122 male, 22 female), age (106 ≤ 39, 8 for 40–60, and 30 for >60 years) and by KL grading (52 grade 0, 9 grade 1, 43 grade 2, 18 grade 3, and 22 grade 4). The minimum number of KL grading was 9. We used the electro-stethoscope that was assembled with a conventional stethoscope for obtaining sound signal. The knee sound signal recorded is shown in Fig. 2, in which the sound signal of multiple cycles is aligned with the goniometer data. For viewing the details of the VAG signal, the ascending and descending limbs are amplified and shown in Fig. 3a, b.

**Fig. 4:**
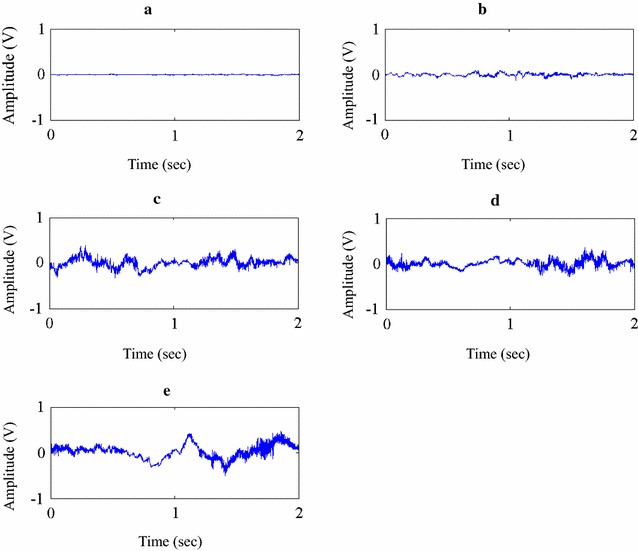
Representative signal for grade 0–4 is shown in **a**–**e**. The sample sound signal is output to the speaker with a cycle of 2 s and lasting for 20 s. The quality of this simulation sound signal is quite like the real-world signal

The knee sound signal database was built with all the collected sound signals. Every sound signal was associated with an OA stage classified by KL grading obtained by X-ray. A wavelet transform of the signal is performed for each KL category. The median of the wavelet coefficient is selected in each KL category. Then, the inverse wavelet transform is carried out to obtain the synthesis signal. The synthesis sound signal is shown in Fig. 4a–e, corresponding to KL grade 0–4. The sounds coming from the medial or lateral compartment of the knee could be adequately assessed with this approach using the stethoscope on the patella. The method using the stethoscope that was an innovative device with a goniometer on the patella would be a valid way to assess osteoarthritis in medial and lateral compartments.

In order to compare the median coefficient algorithm, we also synthesized the corresponding signal in each category by mathematical means. Both types of synthesis signal were then evaluated by three independent physicians for quality assessment. Three clinicians assessed the sounds and rated them. Raters were 47, 47, and 52 years old, and had 20, 20, and 25 years of clinical experience. All three are board-certified orthopedic surgeons. The quality assessment was recorded as a Likert score, with 0 indicating the worst and 5 indicating the best. The descriptive statistics for the Likert score are shown in Table 1. Because the minimum number of KL grading is 9, N is 9. The Wilcoxon signed-rank test is shown in Table 2 (N = 9). Significant results are shown in Table 3. All five categories achieve a statistically significant difference with p < .05.

**Table 1:** Descriptive statistics for Likert score of two different synthesis signals

	N	Mean	Std. deviation	Minimum	Maximum
KL0_median	9	3.6667	1.00000	2.00	5.00
KL1_median	9	3.4444	.88192	2.00	5.00
KL2_median	9	3.3333	1.11803	2.00	5.00
KL3_median	9	4.3333	.86603	3.00	5.00
KL4_median	9	4.0000	.86603	3.00	5.00
KL0_mean	9	2.2226	.52705	2.00	3.00
KL1_mean	9	1.8889	.78174	1.00	3.00
KL2_mean	9	1.7778	.66667	1.00	3.00
KL3_mean	9	2.1111	.92796	1.00	3.00
KL4_mean	9	2.4444	.52705	2.00	3.00

The minimum number of KL grading is 9

KLX_median: median value for KL grading X

KLX_mean: mean value for KL grading X

**Table 2:** Wilcoxon rank scale test for comparing the two methods

		N	Mean rank	Sum of ranks
KL0_mean-KL0_median	Negative ranks	7^a^	4.79	33.50
	Positive ranks	1^b^	2.50	2.50
	Ties	1^c^		
	Total	9		
KL1_mean-KL1_median	Negative ranks	6^d^	3.50	21.00
	Positive ranks	0^e^	.00	.00
	Ties	3^f^		
	Total	9		
KL2_mean-KL2_median	Negative ranks	7^g^	4.00	28.00
	Positive ranks	0^h^	.00	.00
	Ties	2^i^		
	Total	9		
KL3_mean-KL3_median	Negative ranks	8^j^	4.50	36.00
	Positive ranks	0^k^	.00	.00
	Ties	1^l^		
	Total	9		
KL4_mean-KL4_median	Negative ranks	8^m^	4.50	36.00
	Positive ranks	0^n^	.00	.00
	Ties	1^o^		
	Total	9		

The minimum number of KL grading is 9

^a^KL0_mean < KL0_median

^b^KL0_mean > KL0_median

^c^KL0_mean = KL0_median

^d^KL1_mean < KL1_median

^e^KL1_mean > KL1_median

^f^KL1_mean = KL1_median

^g^KL2_mean < KL2_median

^h^KL2_mean > KL2_median

^i^KL2_mean = KL2_median

^j^KL3_mean < KL3_median

^k^KL3_mean > KL3_median

^l^KL3_mean = KL3_median

^m^KL4_mean < KL4_median

^n^KL4_mean > KL4_median

^o^KL4_mean = KL4_median

**Table 3:** Test statistic by Wilcoxon Rank test for two different methods

	KL0_mean-KL0_median	KL1_mean-KL1_median	KL2_mean-KL2_median	KL3_mean-KL3_median	KL4_mean-KL4_median
Z	−2.226^a^	−2.271^a^	−2.388^a^	−2.533^a^	−2.565^a^
Asymp. Sig. (2-tailed)	.026	.023	.017	.011	.010

^a^Based on positive ranks

^b^Wilcoxon signed ranks test

The sample signals were evaluated using different algorithms (median and mean). The cross-table validation of the synthesis signal with sample signals is shown in Table 4. The main diagonal elements represent the correct classification by the KL divergence metric, while the off-diagonal elements represent the misclassified cases. The number of patients for testing the discriminant power of each KL divergence metric was 150. The accuracy rate of the median coefficient algorithm and mean coefficient algorithm were 93 % and 88 %, respectively. The accuracy rate of the median coefficient algorithm for cross-validation was better than the mean coefficient algorithm for synthesis of VAG as described above.

**Table 4:** Cross-table for testing the discriminant power of the KL divergence metric

True\classified	KL0	KL1	KL2	KL3	KL4	Total
KL0_ median	128	22	0	0	0	150
KL1_ median	12	138	0	0	0	150
KL2_ median	0	4	144	2	0	150
KL3_ median	0	0	4	140	6	150
KL4_ median	0	0	0	5	145	150
KL0_ mean	137	13	0	0	0	150
KL1_ mean	32	116	2	0	0	150
KL2_ mean	0	5	143	2	0	150
KL3_ mean	0	2	8	129	11	150
KL4_ mean	0	0	2	10	138	150

The number of patients for testing the discriminant power of each KL divergence metric is 150

Accuracy rate(median) = .93 (93 %)

Accuracy rate(mean) = .88 (88 %)

## Discussion

OA of the knee is a very common disease that mainly affects the elderly. X-ray remains the gold standard for the diagnosis of OA, and although auscultation of the knee is non-invasive, it is still not widely used in clinical practice. Generated during knee ROM, the crepitus sounds are non-stationary and multi-component in nature, and the optimal signal analysis method remains to be elucidated. However, because of the potentially harmful radiation effects and cost of X-rays, non-invasive auscultation of the knee can provide a useful alternative to conventional routine X-ray examination. The grading of knee OA using X-rays correlated closely with the signal characteristics of VAG.

Using a line-in cable, a traditional microphone was connected to a PC. The microphone was also connected to the plastic tube of a stethoscope. This innovative equipment worked well in getting the sound signal with good quality and available facility. In the process of setting up the equipment for data acquisition, the goniometer accompanying the stethoscope is of crucial importance.

In Fig. 2, the information on knee position and facilitate data segmentation is provided by using the goniometer data. The VAG signal was only retained within 25–75° during the ROM. The information on signals during the start and ending stages were discarded.

In Fig. 3, the VAG signal for knee extension (90–0°) and knee flexion (0–90°) is shown. It can be seen that the two signals have some similarity, but are not identical. During knee ROM for VAG data acquisition, the extension stage was assisted by the examiner’s hand while the patient’s leg muscle partially contracted to counter gravity. However, during knee flexion, the examiner’s hand is more relaxed, because there is no need to counter gravity. At the same time, the patient’s thigh muscle is more relaxed due to the weight of the leg. However, the extension/flexion process shares the same tracking path, i.e., the patella-femoral trochlear and medial/lateral femur condyle. Therefore, in Fig. 3, there is some similarity between the two signals.

In our previous study, we found that the characteristic frequency for differentiating the diseased signal was about 50–200 Hz. Therefore, the VAG signal is first processed with the Fourier transform to obtain the whole frequency spectrum (0–2000 Hz), and then the 50–200 Hz segment is selected and divided into fifteen portions. Each portion consists of 10-Hz-wide signals. Next, an inverse Fourier transform is done to obtain the time-domain signal. Originally, we tried to perform VAG signal synthesis on the Fourier domain. However, we found that it was difficult to obtain a good quality signal due to the variation in the input samples. The difficulties of Fourier transform arise from two aspects: one is the versatility of the 2-second cycle. For each test subject, a VAG signal lasts about 20 s, but the individual cycles are not all the same. If the knee ROM cycle is versatile, then the frequency component will vary accordingly. The other problem comes from the sound signal amplitude variation due to friction noise, surrounding atmosphere noise, and irregularity of the muscle contraction activity.

In order to solve the problem of noise and outliers, the Haar wavelet transform was used to further treat the 50–200 Hz reconstructed signal. We have performed up to twelve levels of Haar transformation to completely decompose the VAG signal. In the Haar wavelet system, the coefficients are all real, and deeper levels of Haar represent the low frequency parts. In contrast, the shallow level of decomposition represents the high frequency parts. However, it is just an analogy to describe the Haar decomposition system using the terms of frequency, because, in fact, the Haar decomposition has its own mathematical meanings.

The merits of Haar decomposition are twofold: the decomposition coefficient is real, instead of complex as in the Fourier analysis. As we know, when performing the median number selection, a delicate problem will be encountered when dealing with complex numbers. On the other hand, we found that the wavelet transform is more robust to the signal noise. In order to obtain a typical signal that represents each KL category, the median of the Haar coefficient is chosen in each wavelet level to do the inverse transformation. This method elegantly resolves the problem of averaging the signal from different patients to obtain a “population mean” in each KL category. As we know, direct averaging of the time-domain signal is not feasible due to the great variation of the input samples. However, just by choosing the median of every level of Haar coefficient, we can obtain a hybrid signal representing the “population mean” in each KL category. For comparison, the mathematical mean of the wavelet coefficient is taken for the inverse transformation. The comparison of these two methods is then assessed by three independent physicians. The signal is fed to the physician in sequence three times, and the quality is recorded using a Likert score as shown in Tables 1 and 2. The Wilcoxon rank test statistic is shown in Fig. 4. Fortunately, all KL grade categories achieve statistical significance (p < .05), i.e., the median synthesis method outperformed the mathematical mean methods.

For testing the classification power of the synthesis VAG signal, the distance measurement is chosen to use the KL divergence. Here, the abbreviation name is the same as the KL grade (Kellegren-Lawrence score), but the different contexts help readers differentiate them. However, it can be differentiated correctly from the related context. In information theory, the KL divergence is a useful metric for assessing the similarity between two probability distribution functions (PDF). In Eq. (4), it is important to keep the synthesis signal over the denominator position, while the testing sample remains in the numerator position. The KL divergence metric is asymmetric, but the numerator/denominator position cannot be changed. After calculating the KL divergence distance, the nearest neighbor rule (NN) is used to assign the KL categories. The cross-validation result is shown in Table 4. The accuracy rate of the median was 93 % and the accuracy rate of the mean was 88 %. However, from Table 4, it can be observed that there is some overlap between the neighboring KL grades as judged by the synthesis signal classifier. This is reasonable because the similarity between neighboring KL grades is high. The sound of KL grade 4 feels like grade 3, not like grade 2, and can in no way be judged as grade 0. Similarly, the grade 1 signal is sometimes judged as grade 0, but it cannot be classified as grade 4. In our experience, the human learning capability is great. Once a person is familiar with the synthesis signal, they can perform the classifying task by touch feeling precisely, with little error. Therefore, the artificial signal we synthesized has the potential to build a learning system for medical students, internships, and para-medical personnel.

## Conclusion

In conclusion, an innovation stethoscope device for use with a goniometer obtains knee sound signals. Inputted to a PC, the sound signals were segmented according to the goniometer data. A Fourier transform of the signals was performed over the correlated frequency segment. Implementations of the inverse Fourier transform in order to get a time-domain signal. Haar wavelet transformation is then carried out. Coefficient medians are chosen to inverse transform the synthesis signal. The quality of synthesis signal was assessed by clinicians and was found to be better than the mean method. Cross-validation results were good and achieved a 93 % accuracy rate. Therefore, our method provides a feasible way to evaluate crepitus sounds that may assist in the diagnosis of knee OA.

## Abbreviations

VAG: vibroarthrographic
OA: osteoarthritis
KL grade: Kellgren–Lawrence grade
CT: computed tomography
MRI: magnetic resonance imaging
ROM: range of motion
PF: patellofemoral
PC: personal computer
KL divergence: Kullback–Leibler divergence
PDF: probability distribution functions
NN: neighbor rule
